# How Effective Are Forecasting Models in Predicting Effects of Exoskeletons on Fatigue Progression?

**DOI:** 10.3390/s24185971

**Published:** 2024-09-14

**Authors:** Pranav Madhav Kuber, Abhineet Rajendra Kulkarni, Ehsan Rashedi

**Affiliations:** 1 Department of Industrial and Systems Engineering, Rochester Institute of Technology, Rochester, NY 14623, USA; pmk2015@rit.edu; 2 Department of Computer & Information Science & Engineering, University of Florida, Gainesville, FL 32611, USA; abhineet.kulkarni@gmail.com

**Keywords:** ergonomics, industrial exoskeletons, human muscle fatigue, forecasting, machine learning, workload monitoring system, workplace safety

## Abstract

Forecasting can be utilized to predict future trends in physiological demands, which can be beneficial for developing effective interventions. This study implemented forecasting models to predict fatigue level progression when performing exoskeleton (EXO)-assisted tasks. Specifically, perceived and muscle activity data were utilized from nine recruited participants who performed 45° trunk flexion tasks intermittently with and without assistance until they reached medium-high exertion in the low-back region. Two forecasting algorithms, Autoregressive Integrated Moving Average (ARIMA) and Facebook Prophet, were implemented using perceived fatigue levels alone, and with external features of low-back muscle activity. Findings showed that univariate models without external features performed better with the Prophet model having the lowest mean (SD) of root mean squared error (RMSE) across participants of 0.62 (0.24) and 0.67 (0.29) with and without EXO-assisted tasks, respectively. Temporal effects of BSIE on delaying fatigue progression were then evaluated by forecasting back fatigue up to 20 trials. The slope of fatigue progression for 20 trials without assistance was ~48–52% higher vs. with assistance. Median benefits of 54% and 43% were observed for ARIMA (with external features) and Prophet algorithms, respectively. This study demonstrates some potential applications for forecasting models for workforce health monitoring, intervention assessment, and injury prevention.

## 1. Introduction

Predicting trends in physiological conditions when performing tasks can be insightful for injury prevention, especially in industrial settings where physical demands often exceed human capabilities. Even with increasing industrial automation, work-related musculoskeletal disorders (WMSDs) remain a workplace epidemic across the world as humans are expected to match the performance of robots that do not get fatigued. In contrast, workers frequently engage in repetitive tasks leading to fatigue and overexertion, posing significant safety risks [1]. In such cases, interventions to improve workplace health and safety are deployed, consisting of ergonomic controls [2,3], workforce training [4], and assistive devices [5]. The field of industrial ergonomics revolves around evaluating the effects of such interventions to identify their fit for the task and the worker. Commonly, such evaluations include comparative assessments between different interventions and control conditions, using both subjective/perceived measures and objective measures obtained using sensor technology [6]. Typical indicators for studying physical exertion levels include changes in body posture/kinematics [7], muscle activity [8], metabolic rate [9], balance [10] and kinetics [11]. In addition, measures that capture the temporal or cumulative effects of task performance, such as fatigue, are particularly important. Fatigue is defined as the decline in an individual’s ability to sustain necessary performance capabilities and estimating fatigue progression provides a valuable metric for assessing the impact of interventions and understanding their long-term benefits [12,13]. Fatigue can impair an individual’s ability to function effectively by adversely affecting cognitive (affecting decision making abilities and slowing reactions) and physical capabilities (strength/endurance) [14], leading to reduced performance [15] and safety.

Most industrial tasks are repetitive in nature, lead to fatigue among human workers when performed for long durations and can lead to injury due to overexertion. Industrial variants of exoskeletons (EXOs), especially passively actuated ones, may benefit such personnel by reducing required muscle effort during tasks in injury prone regions of the body (such as shoulder, and low-back) and ultimately improve workplace safety [16]. Prior studies have shown that EXOs can reduce injury risk by decreasing the physical demands imposed on workers [17,18,19]. The effects of using EXOs on the human body have been evaluated through evaluation-based studies, and the findings have indicated improved endurance of up to 60% as well as performance up to 85% [20,21,22] with up to 65% reduction in muscle demands [20,21,23,24,25,26,27]. One categorization of industrial EXOs is based on the body region they assist, including lower-back, shoulder, and legs. As per their name, back-support industrial EXOs (BSIEs) are designed to provide benefits in tasks that include performing trunk bending [21,28,29,30], such as when performing assembly tasks, or when reaching/lifting items [9,31,32,33,34,35,36]. Recent studies have highlighted current challenges in evaluating their effects in real-world environments, i.e., during field testing for proper implementation of the assistive devices [37]. To support effective evaluation and implementation, there is a need to develop data-driven approaches for assessment of the effects of the devices on their wearers. Prior work has been undertaken towards similar goals, where machine learning models were developed to detect the presence of fatigue and their levels [38,39], which can determine the demands imposed on workers when performing tasks while assisted with a BSIE.

Forecasting models offer significant advantages for predicting future events compared to traditional machine learning approaches or calculating differences in means across recorded measures [40,41]. Primarily, forecasting models consider trends over time, which can enable the prediction of not only the amount of increase in demands, but the time intervals for increase in their levels. This can also account for nonlinearity in increase/decrease in the physiological signals [42,43]. Machine learning models focus on static patterns, and they often require extensive feature engineering to handle time-related information effectively [12]. Forecasting models can be more effective in time-series prediction, and can also integrate external factors, like environmental conditions and individual worker demographics, enhancing prediction accuracy [44]. In contrast, the traditional approach of comparing differences in means across study conditions focuses on past patterns and anomalies without offering proactive insights for future outcomes. Common forecasting models include time series analysis, regression models, machine learning algorithms, and neural networks [12,45,46,47]. These models can predict trends, or an increase/decrease in physical and cognitive capacity of the human body. For instance, prior studies have implemented such forecasting models to predict movement [40], muscle activity [43], blood pressure [48], cybersickness [46], stress [49] and neuromuscular fatigue [42,44]. Besides providing early warnings, forecasting can support comparative assessments of interventions over longer periods of time.

Forecasting models have demonstrated capabilities in predicting temporal progression in measures of muscle activity and fatigue, which can be used to develop wearable devices to prevent back-injury. For instance, a recent study showed that forecasting of features collected from surface electromyography (EMG) for a 25 s horizon led to a mean absolute error of 6.88% [43]. Forecasting has also been implemented to improve adaptive assistance from EXOs by accounting for changes in muscle activity features with fatigue progression. In one study, models for predicting lower-limb muscle fatigue were developed, with accuracies of RMSE values ~4% to account for changes in EMG signals with fatigue and achieve compliance with an EXO [50]. Similarly, another study implemented forecasting methods to predict muscle fatigue indices to provide effective adaptive control of EXOs. Outcomes of the study showed that models could forecast time and frequency domain indices of muscle fatigue ~22.6 s and 60 timesteps in the future with a MAE of ~16% during voluntary contractions [51]. While muscle fatigue measures are beneficial for understanding physical demands in specific muscle locations, perceived measures of fatigue can provide a more realistic understanding of the demands experienced by an individual. One prior study showed that forecasting models can predict perceived levels of physical demand using subjective exertion ratings and motion data during walking tasks with MAE values of <1.24 [44]. A model implemented in another study was able to predict perceived fatigue using wearable sensors up to 80 timesteps ahead with an accuracy of ~80% and a co-relation of 0.92 [42]. A similar approach can also be implemented to predict future states of perceived fatigue and its progression when performing tasks with an EXO.

The objectives of this study were to develop a reliable forecasting model for predicting future trends in perceived fatigue levels in the back and leg regions of workers by implementing multiple forecasting algorithms, both with and without features extracted from wearable sensors that recorded muscle activity. For this purpose, our study encompassed a controlled experiment, where study participants performed repeated trunk flexion tasks once with and then without being assisted by a BSIE. The secondary objective included comparing forecasted fatigue level between with vs. without assistance conditions to understand temporal benefits provided by BSIEs. This study presents a novel approach of utilizing forecasting models for predicting benefits in fatigue progression provided by BSIEs. Ultimately, the efforts presented in this study aim to improve worker safety, productivity, and reduce injury rates by developing a system to further understand how EXOs impact fatigue over time.

## 2. Materials and Methods

### 2.1. Study Participants

For developing forecasting modes, an experiment was conducted by recruiting 9 adult male university students with moderate exercise habits (twice per week) and a lack of musculoskeletal disorders in the past six months. Anthropometric dimensions measured consisted of age in years, height in cm, weight in kg, and body-mass index as shown in Table 1. Consent was obtained in written form, approved by the review Board (HSRO#01113021) with protocols designed according to the tenets of the Declaration of Helsinki.

### 2.2. Experimental Apparatus and Equipment

This study incorporated a 2 × 2 experimental design with independent factors of assistance (with/without BSIE) and postural asymmetry (no asymmetry/45° postural asymmetry). Tasks included performing 30 s bouts of maintaining trunk flexion at a ~45° sagittal flexion angle preceded and succeeded by 15 s of standing still, performed intermittently with 15 s intervals of relaxation. Durations were adjusted based on pilot experimental runs.

Among the various types of BSIEs, passive devices are more likely to be adopted due to their ease of use, and compact and lightweight structure. As such passive devices are actuated with mechanical/pseudo-mechanical actuators that provide ~20–30 lbs of assistance to their wearers [22,52], these devices are more beneficial in providing cumulative benefits and understanding temporal effects of such devices can be insightful. Thus, we selected a passive rigid BSIE named as the BackX Model AC (SuitX, Emeryville, CA, USA). Throughout the experiment, the device was set at the medium assistance level (~25 lbs).

**Figure 1:**
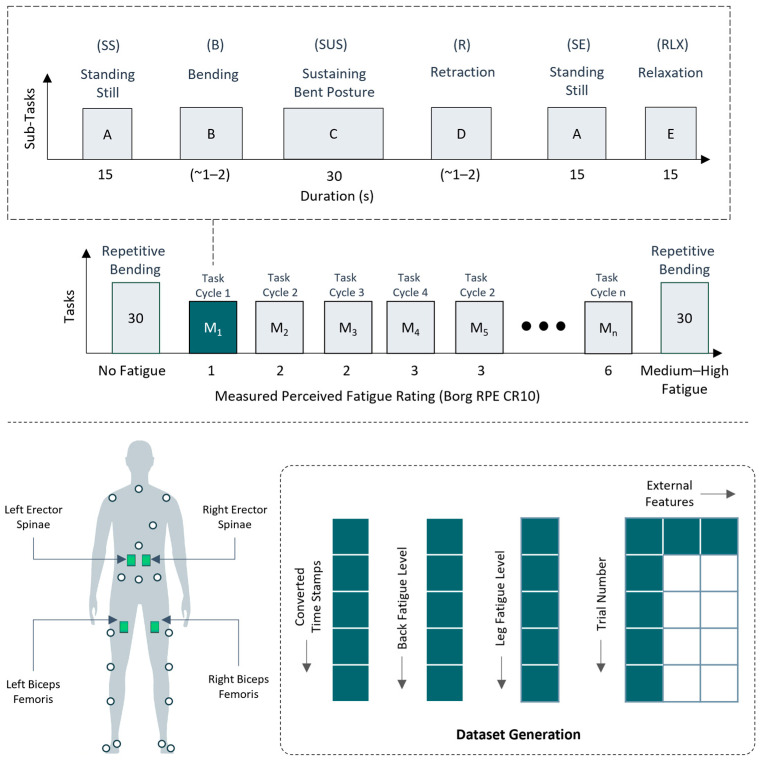
Illustration depicting experimental tasks of repetitive and intermittent trunk flexion task cycles and activities of standing still (SS), bending (B), sustaining bent posture (SUS), retraction (R), and relaxation performed within each task.

Both local and global measures of fatigue in back and leg regions were recorded throughout the study. Localized muscle fatigue was recorded using surface electromyography (EMG). As shown in Figure 1, Trigno Wireless EMG sensors (Delsys, Natick, MA, USA) with sampling frequency of 1200 Hz were placed on the left/right erector spinae longissimus (LES/RES) and the biceps femoris muscles (LBF/RBF). On the other hand, global (perceived) fatigue was measured using ratings of perceived exertion (RPE) on the Borg RPE CR-10 scale [6,53], with levels 0, 1, 2, 3, 4, 5, 7, 10 indicating ‘no exertion’, ‘very slight’, ‘slight’, ‘moderate’, ‘somewhat severe’, ‘severe’, ‘very severe’ and ‘maximal’ levels of fatigue, respectively.

### 2.3. Experimental Design and Procedure

The experiment used to develop models was the same as described in detail in our previously published study [38], where the complete experiment involved performing three sessions (training/session-1, session-2, session-3) with a 2-day break for muscle recovery. Participants were instructed to perform a wall-sit task for self-calibration of their perceived fatigue ratings in the legs using the Borg RPE CR-10 scale. Subsequently, maximum voluntary contractions (MVCs) were recorded from all four muscles (LES, RES, LBF, and RBF), which were used later for normalization purposes. Each of the two subsequent experimental sessions included performing bending tasks with/without the BSIE in symmetric and asymmetric postures. Participants performed tasks in both postures with and without assistance in each of the two sessions in a counterbalanced order across participants. For each experimental condition (with/without assistance and with/without asymmetry), a trial included participants performing intermittent task cycles of 30 s sustained bending, and two 15 s standing still activities with 15 s relaxation breaks until they reached a medium–high fatigue perceived exertion level on the Borg scale representing a number 7/10 (Figure 1). Repetitive trunk bending was performed at the beginning and end of each session.

### 2.4. Signal Processing and Dataset Generation

**Figure 2:**
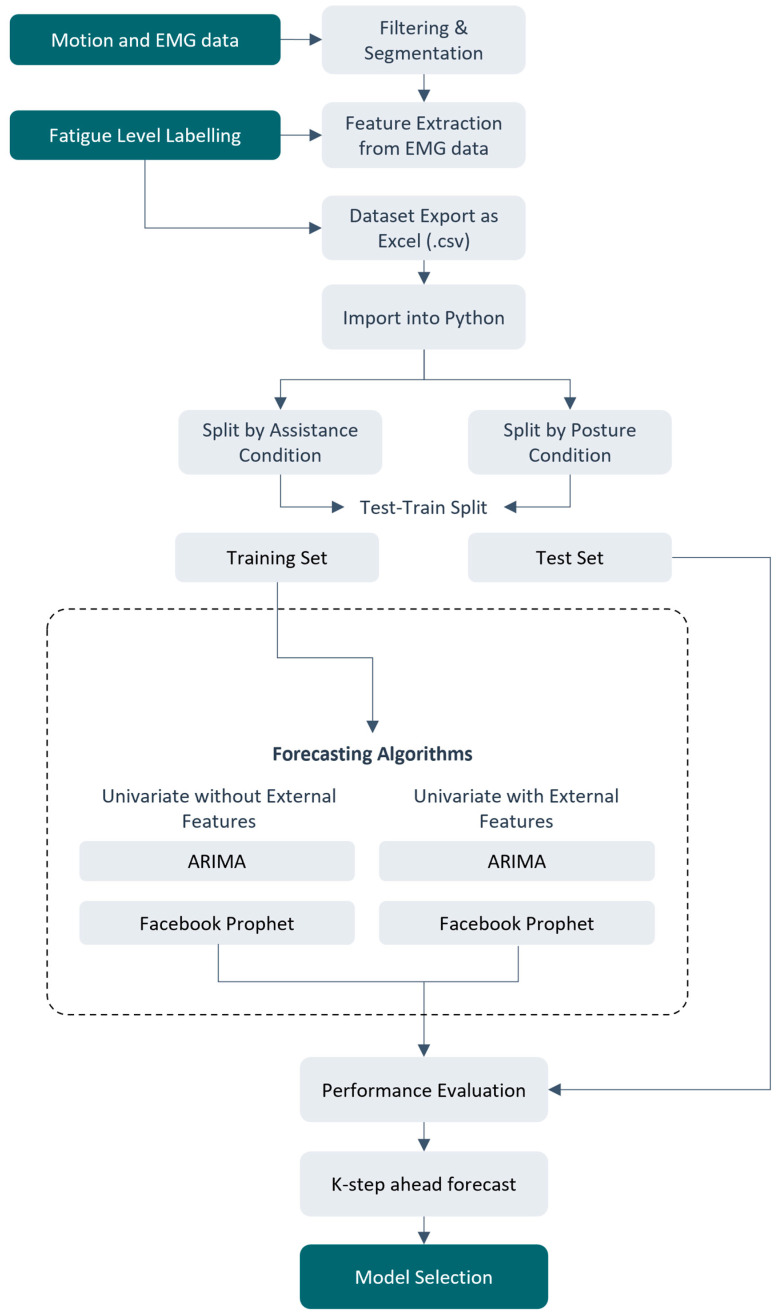
A flowchart depicting model development and testing procedure for model selection.

The data collected during experiments consisted of subjective (perceived fatigue levels) and objective (EMG sensor data) for each experimental trial. Perceived fatigue levels were recorded and noted by investigators during the relaxation period after each 60 s trial. Furthermore, EMG data were analyzed using a MATLAB code, which imported and segmented EMG data into distinct portions based on the type of activity (standing still at start/end, sustaining trunk flexion, and bending/retraction) as shown in Figure 2. Upon segmentation, the EMG signal was first filtered using a Butterworth filter in the 30 Hz and 300 Hz band to remove noise. As the raw EMG data cannot be directly used for processing, correlation, or comparison, we calculated the root-mean-square (RMS) of the signal. Features extracted from the RMS included peak and mean amplitude of muscle activity for each of the tasks within each task cycle. Both peak and mean amplitude of the signal were normalized based on peak values obtained during MVC trials. In addition, the segmented time-series data was also converted to the frequency domain using the fast Fourier transform (FFT) to calculate the median frequency of the signal. The final generated dataset included fatigue levels in the low-back region and a total of 12 features of peak and mean amplitude and median frequency extracted from EMG sensors on the left and right erector spinae muscle, which represented their respective experiment trial number (Table 2). Features were selected based on the most important features identified in our previous study [38]. While the complete study included both asymmetric and symmetric posture conditions, this study only considered data for the symmetric posture condition and consisted of performing repetitive intermittent trunk flexion task cycles as previously described with and without assistance.

### 2.5. Forecasting Models

Two of the most common forecasting models include ARIMA (Autoregressive Integrated Moving Average) and Facebook Prophet, both of which are well-regarded for their efficacy in time series forecasting [44,54]. ARIMA models are effective when the data show clear temporal structures and patterns, such as trends and cycles and have been extensively used due to their flexibility and the interpretability of their parameters. Facebook Prophet is an open-source tool designed for forecasting time series data and temporal patterns. Prophet is known to handle missing data and large outliers robustly and can be efficient for predicting data that is generated at predetermined specific time intervals. While both ARIMA and Prophet are powerful tools for time series forecasting, their suitability depends on the specific characteristics of the data and the forecasting objectives. ARIMA is highly effective for stationary time series data with minimal seasonal variation, offering precise control over model parameters. On the other hand, Prophet excels in handling complex seasonal patterns and incorporating external factors, providing a flexible and user-friendly framework for forecasting.

#### 2.5.1. Univariate ARIMA Model

The ARIMA model is expressed as ARIMA (p, d, q), where p is the number of lag observations included in the model (autoregressive part), d is the number of times that the raw observations are differenced to achieve stationarity (integrated part), and q is the size of the moving average window. ARIMA models combine three components:

- Autoregressive (AR) portion: Uses the dependency between an observation and lagged observations. For example, AR (1) means the current value depends on the previous value.
- Integrated (I) portion: Involves differentiating the data to make it stationary, meaning the mean and variance are constant over time. Differencing is subtracting the previous observation from the current observation.
- Moving Average (MA) portion: Models the error term as a linear combination of past error terms. For example, MA (1) means the current error term depends on the previous error term.

The model works by fitting these components to the historical data and using them to forecast future values. To determine the correct order of all three of the above components for each study participant, we used the automatic ARIMA method [55]. These parameters were used to forecast perceived fatigue levels in the low-back region.

#### 2.5.2. Univariate Prophet Model

Prophet is an additive model designed for time series forecasting, especially those with strong seasonal effects and missing data. It decomposes the time series into three main components:

- Trend: Captures long-term increase or decrease in the data.
- Seasonality: Captures periodic changes (daily, weekly, yearly).
- Holidays: Captures effects of holidays and special events.

Prophet fits these components to the historical data using a combination of curve fitting and Bayesian methods, allowing it to handle outliers and missing data effectively. The reason Prophet model was chosen was due to its ability and robustness to handle outliers efficiently [54].

#### 2.5.3. Univariate ARIMA Model with External Features

This model extends the ARIMA framework by including external variables. These external variables, or exogenous variables, are incorporated into the model to account for their influence on the forecasted variable. The ARIMAX model is specified as a combination of traditional ARIMA model, and a linear regression defined on the external features.

#### 2.5.4. Univariate Prophet Model with External Features

Prophet has an advantage over the ARIMA model by also considering nonlinear relationships with the external features. In addition, the Prophet model with external features may be beneficial for the interpretation of the outcomes.

### 2.6. Model Development Procedure

The generated dataset in the form of a single excel spreadsheet was imported into Python v3.9 software (Wilmington, DE, USA). After importing, separate subgroups were created for each participant and were categorized as with/without assistance. For this study, we only considered symmetric bending postures, and data collected during asymmetric trunk bending were excluded. This was followed by conversion of the trials into timestamps (1 trial corresponding to 60 s + 15 s rest = 1.25 min). For each participant, we segmented the number of rows into a holdout set (last 20% of the data) used for evaluating the models (test set). For models that did not use external features, there was no need to lag as these models were trained using perceived fatigue data only. The next step consisted of model training and performance evaluation. On the other hand, for models that used external features, the external features were lagged by 4-time steps to account for (a) test set needing external feature values during performance evaluation, and (b) to account for potential delays from a cold start when using the system in the real world.

### 2.7. Forecast Performance and Comparative Assessments

The performance of forecasting perceived back fatigue levels was evaluated by calculating the root-mean-square error (RMSE) for each of the four models and for all the study participants. Specifically, to compare across models, perceived back fatigue levels were predicted for four-steps ahead of the back fatigue recorded during the last trial for both with and without assistance conditions. Outcomes were reported as mean, maximum, minimum, and standard deviation in the RMSE values for all participants; they were also reported for each participant separately. In addition, comparative assessments were conducted for with vs. without assistance conditions by forecasting fatigue levels up to 20 trials (representing a duration of 25 min). We decided to select 20 trials by considering the mean number of trials completed which amounted to ~15 trials. Thus, perceived back fatigue levels were compared with vs. without assistance conditions at 20 trials by calculating the percentage difference between the levels with positive percentages indicating benefits in favor of assistance condition. Similarly, fatigue levels for both assistance conditions were plotted along with their forecasted values up to 20 trials to determine high performing models as well as to visualize the effects of the BSIE in delaying fatigue progression.

## 3. Results

The outcomes of our analysis can be categorized into two subsections, first where performance of forecasting models was evaluated, and second where forecasting models were implemented to evaluate effects of using assistance provided by a BSIE on fatigue progression.

### 3.1. Performance Evaluation of Forecasting Models

**Figure 3:**
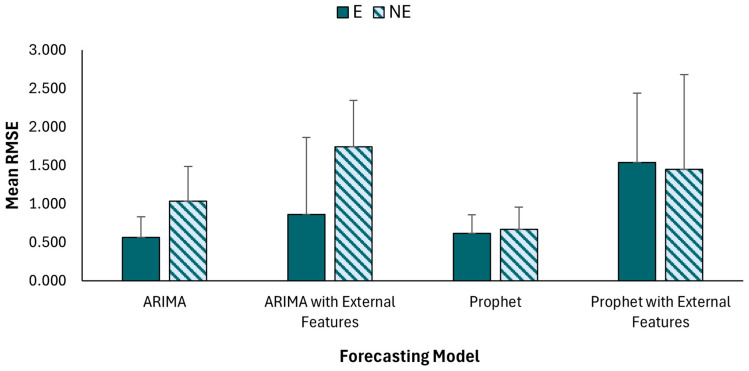
Comparison of mean Root Mean Square Error (RMSE) values for each of the developed forecasting models across with (E) assistance and without (NE) assistance conditions.

The overall performance of each of the four forecasting models, namely ARIMA and Prophet with and without external features, was assessed with mean and spread in the obtained RMSE values for forecasting 4 steps, which can be viewed in Figure 3. The lowest mean RMSE values were obtained when external features were not used. In addition, ARIMA models resulted in ~90–100% lower mean RMSE values during trials where BSIE assistance was used vs. without assistance conditions. On the other hand, Prophet models displayed similar results between with and without assistance conditions. The ranges of the RMSE values can be viewed in Table 3 showing maximum values for Prophet model when used with external features. Table 4 denotes the variation in RMSE values for forecasting fatigue levels for each session in this study.

### 3.2. Comparison of Exoskeleton Effects with Forecasted Fatigue Levels

**Figure 4:**
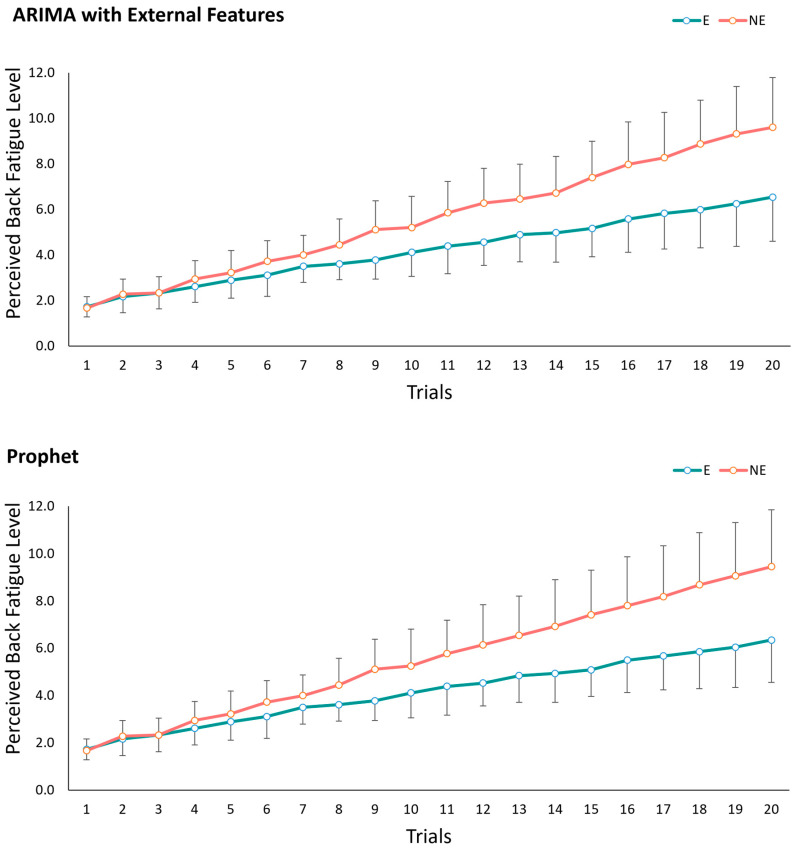
Comparison of mean values of perceived back fatigue levels across all participants for with vs. without assistance conditions for best performing models of (**top**) ARIMA with external features and (**bottom**) Prophet across 20 trials with forecasting.

**Figure 5:**
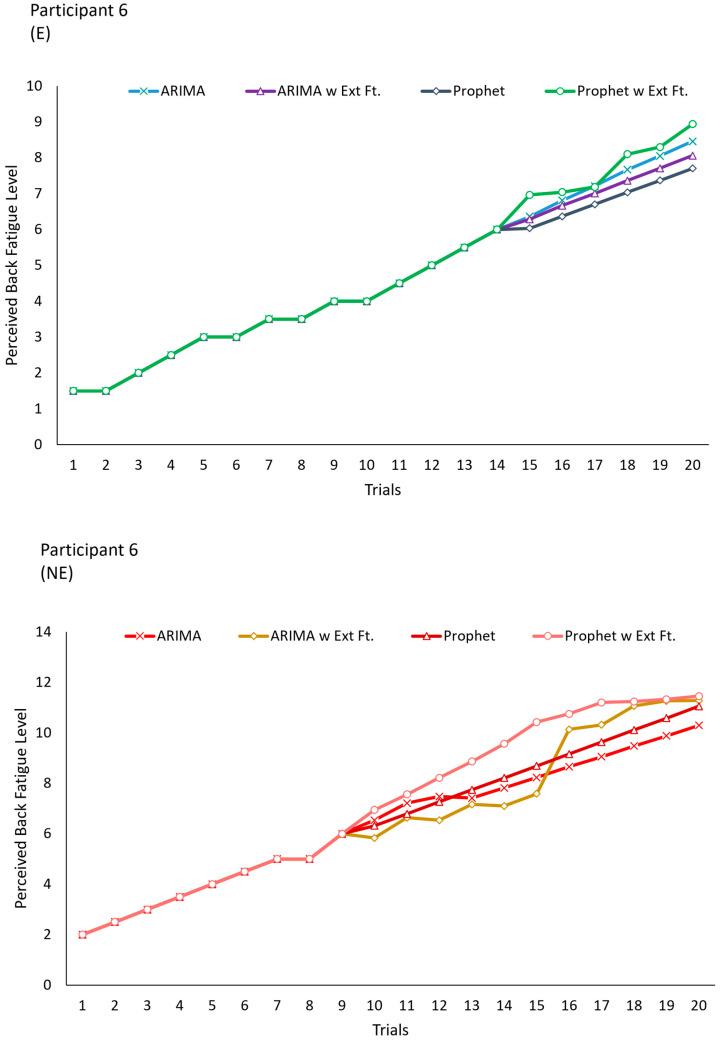
Comparison of perceived back fatigue levels across different forecasting models for (**top**) with denoted by E and (**bottom**) without assistance denoted by NE conditions for a single participant.

**Figure 6:**
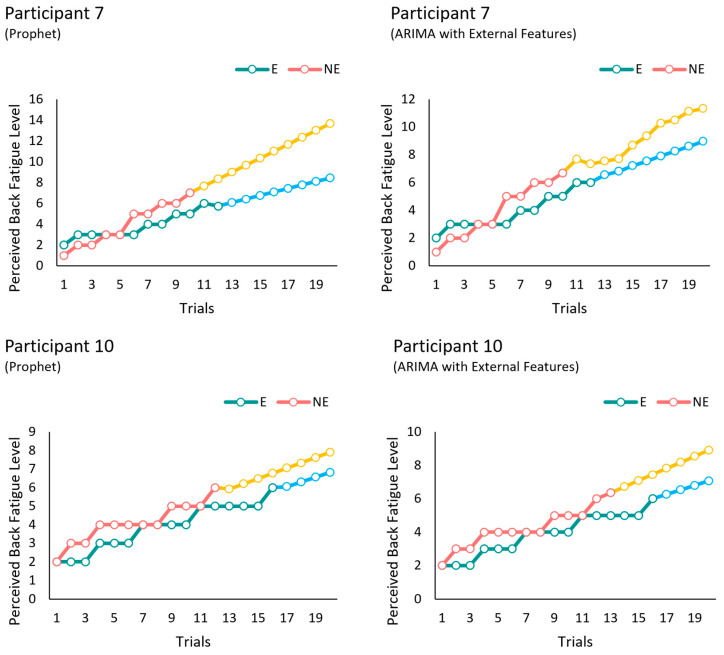
Comparison of mean values of perceived back fatigue levels across all participants for with (E) vs. without (NE) assistance conditions for best performing models of (**top**) ARIMA with external features and (**bottom**) Prophet across 20 trials with forecasting (yellow and blue colors indicate forecasted fatigue levels for without and with assistance conditions respectively).

In the second portion of our analysis, perceived fatigue in the low-back region was forecasted for up to 20 trials, and the benefits were evaluated at 20 trials by comparing fatigue levels between with and without BSIE assistance conditions. Figure 4 denotes the mean and SD of perceived fatigue levels and their variation with and without assistance for the two best performing models of ARIMA with external features, and Prophet. The slope of the line without assistance was 0.41 and was ~48% higher than the slope of the line with assistance (0.25) when using ARIMA with external features. Similarly, the slope of the line without assistance was 0.41 and was ~52% higher than the slope of the line with assistance which was 0.244. The deviation in the values across participants increased with the number of trials as seen in Figure 4. Meanwhile, Figure 5 depicts differences in predicting fatigue progression for a single participant (#6) when performing tasks with and without assistance with the BSIE. Similarly, progression of fatigue level with vs. without assistance for 20 trials has been displayed for another participant (#7) in Figure 6 along with differences caused by the type of model used. Differences between with and without assistance conditions were then compared to determine percent benefits of BSIEs in reducing fatigue. As shown in Table 5, benefits of BSIEs were 55% and 51% for the model developed with ARIMA with external features and Prophet model respectively.

## 4. Discussion

This study involved developing forecasting models for predicting perceived fatigue levels in the low-back region when performing EXO-assisted repetitive trunk bending tasks. The efforts in this study were aligned towards developing efficient fatigue detection and monitoring systems for evaluating physical demands in industrial workspaces. The novelty of this study is that this study (a) utilized forecasting models for predicting future levels of perceived fatigue when performing BSIE-assisted tasks, and (b) quantified benefits provided by BSIEs in reducing fatigue progression using the developed models.

Prior studies on evaluating effects of BSIEs on the human body have shown that using these devices can reduce low-back muscle activity up to 40% when performing trunk bending and lifting tasks [56]. These studies often involved participants performing tasks over short durations in a single task (such as sustaining a posture), while field studies demonstrate mixed outcomes possible due to uncontrolled body movements [37]. On the other hand, we incorporated an intermittent task with a range of fundamental body movements (bending/retraction, sustaining bent posture, and static standing), thus enabling simulation of realistic condition in a controlled environment. Moreover, while localized measures, such as muscle activity, can be beneficial in detecting effects on a specific region, perceived fatigue is a global measure that denotes a combination of both the physical and cognitive demands imposed on an individual. Thus, temporal effects of BSIEs, such as changes in fatigue progression, have not yet been explored in sufficient detail. This study provides an effective approach for forecasting demands to assist in understanding long-term impacts of exoskeletons by enabling researchers to predict future trends in fatigue ahead of time. Besides enabling early intervention by ergonomics professionals, this approach can also offset the current limitations of lab-based studies. These include the use of multi-sensor measuring equipment, extensive resources, and practical limitations in conducting long-term and longitudinal studies [37]. In such cases, forecasting methods as demonstrated in this study, can be implemented to provide a more accessible evaluation of perceived physical demands.

Only a few studies were found to have used forecasting models for predicting perceived fatigue [42,44]. The performance of predicting fatigue levels was assessed with similar measures to those used in this study. For instance, mean absolute error (MAE) values of <1.22 indicated the best performing model for predicting fatigue levels using wearable motion sensors [44]. Another study found that the developed forecasting models were able to predict perceived fatigue up to 80-time steps ahead with an accuracy of ~80% and a co-relation of 0.92 [42]. Results in the form of RMSE values obtained in this study ranged from ~0.5 to ~1.7 with higher values observed for without assistance condition. Our findings indicate that ARIMA with external features and Facebook Prophet can be beneficial in forecasting perceived fatigue levels. Particularly, ARIMA models led to ~100% decrease in mean RMSE values with vs. without assistance from BSIE (Figure 3). Higher values for the without assistance condition could be attributed to the lower number of available datapoints for training the models as study participants performed a smaller number of trials without assistance. When performing the tasks with assistance, participants were assisted during sustained bending postures, which enhanced their endurance, leading to higher completed trials, and more data for model training. On the other hand, Prophet models displayed similar results between with and without assistance conditions. This indicates that Prophet models were more resilient to outliers and thus not affected by the lesser amount of data available.

Results of our comparative assessments showed that fatigue progression when assisted with BSIE was slower than when the device was not used. Prior studies have evaluated endurance by measuring time to fatigue during sustained postures. For example, use of a BSIE increased time of sustaining a flexed trunk posture from 3.2 to 9.2 min [30]. In another study, the reduction in low-back muscle activity, heart rate and subjective perspectives indicated improved capabilities during both controlled lab-based and field testing of BSIEs during prone positioning tasks [57]. By considering changes in perceived fatigue levels, this study aimed to obtain a more realistic measure of the effects of using BSIEs. The outcomes of our study showed that respective benefits for each participant at the end of 20 trials were 55% and 51% for ARIMA with external features and Prophet models, respectively. However, as seen in Figure 4, the benefits are expected to steadily increase as the number of trials increases. Specifically, values of slope of fatigue progression line were ~48–52% lower when assisted by the BSIE indicating increased endurance in wearers of these devices. Thus, one of the advantages of the methods implemented in this study is that the trained models can predict future fatigue states provided initial fatigue level data is available. This can then be used to develop guidelines for implementing these devices, decide work–rest ratios, and define shift durations that can be unique to EXO-assisted tasks.

Even though this study implemented forecasting models to predict perceived back fatigue levels, the study was subject to a few limitations that should be recognized prior to interpretation and generalization of the presented outcomes. First, this study was conducted in a laboratory environment where study participants performed highly controlled body movements. On the other hand, real-world tasks may involve complex activities and any such variations within and across the task cycles may decrease prediction performance of the models. However, in such cases, models that include external features from sensors, such as the EMG sensors used in this study can be useful to provide more accurate predictions. It should be noted that our sample size was relatively small with only adult male participants that were recruited from a university student population having a mean age of ~20 yrs. Future studies should incorporate larger sample sizes with diverse population demographics to gain more generalizable model capabilities in the real world. Due to limitations on collected data, we were only able to predict up to 20 time steps since predicting more steps in the future could have led to higher errors. Thus, future work can include a much more comprehensive and larger dataset. Future work would also include improving the overall accuracy of the models. As the proposed forecasting models use collected/recorded data to predict future states, forecasting models can be a suitable alterative to predict individualized/personalized fatigue predictions rather than relying on predefined statistical models and theoretical equations. Future work can also utilize more complex models like long short-term memory (LSTM) neural networks [49]. While external features used in this study were only limited to muscle activity, motion and whole-body stability data was also recorded and future studies can determine sensor systems that are more efficient at improving forecasted fatigue predictions. Our next steps would consider testing the performance of these models in less controlled field studies to determine prediction differences.

## 5. Conclusions

This study demonstrated a novel method of predicting the effects of EXOs on perceived fatigue levels using forecasting models. The data used for developing the models was collected from a controlled laboratory experiment that was conducted by recruiting participants who performed repeated intermittent trunk flexion tasks. The collected perceived fatigue and muscle activity data in the low-back regions were then utilized to develop forecasting models using ARIMA and Prophet algorithms using perceived fatigue levels alone, and then using perceived fatigue levels and features extracted from recorded low-back muscle activity. The analysis was performed in two phases: (a) performance evaluation of models, and (b) comparison of fatigue progression with vs. without assistance conditions.

Performance evaluation outcomes demonstrated that univariate models without external features performed better than those with external features of muscle activity measures. The ARIMA model led to the lowest mean (SD) of RMSE of 0.56 (0.26) for data collected when assistance was provided with a BSIE. On the other hand, Prophet model without external features led to lowest mean (SD) of RMSE for both assistance conditions and were 0.62 (0.24) and 0.67 (0.29) during with and without EXO-assisted tasks, respectively. Findings from comparative analysis between assistance conditions showed that considering all participants, the slope of the line representing fatigue progression for 20 trials without assistance was ~48–52% more than the slope of the fatigue progression with assistance using ARIMA with external features and Prophet models. The observed reduction in fatigue progression with BSIE, with up to a 54% lower fatigue for the ARIMA model and a 43% lower fatigue for the Prophet model, underscores the potential of these forecasting techniques in optimizing task performance and enhancing the implementation of exoskeleton systems. This study paves the way for future studies to develop efficient fatigue monitoring systems as well as their broader applications in improving assistive technologies and overall industrial ergonomics.

## Figures and Tables

**Table 1:** Mean, standard deviation (SD), and ranges (expressed as minimum–maximum) of anthropometric dimensions of age, height, weight, and body-mass index of study participants.

Demographic Factor	Mean (SD)	Range
Age in yrs.	20.8 (2.8)	18–28
Height in cm	180 (3.09)	176–186
Weight in kg	72.3 (5.7)	65.68–82.44
Body-Mass Index in kg/m^2^	22.19 (2.2)	19.42–26.14

**Table 2:** Features extracted from electromyography (EMG sensors located on left/right erector spinae muscles (LES/RES) along with their respective descriptions.

No.	Parameter	Description
1	nAmpEMGs3_SUS	Normalized peak amplitude in RES during sustained bending
2	nAmpEMGs1_R	Normalized peak amplitude in LES during retraction
3	nAmpEMGs3_R	Normalized peak amplitude in RES during retraction
4	nAmpEMGs1_SE	Normalized peak amplitude in LES during standing at the end
5	nMeanEMGs1_SS	Normalized mean amplitude in LES during standing at the start
6	nMeanEMGs3_SS	Normalized mean amplitude in RES during standing at the start
7	nMeanEMGs1_B	Normalized mean amplitude in LES during bending
8	nMeanEMGs1_R	Normalized mean amplitude in LES during retraction
9	nMeanEMGs1_SE	Normalized mean amplitude in LES during standing at the end
10	nMeanEMGs3_SE	Normalized mean amplitude in RES during standing at the end
11	n_MedianFreqs1_R	Normalized median frequency in LES during retraction
12	n_MedianFreqs3_R	Normalized median frequency in RES during retraction

**Table 3:** Mean, ranges (maximum, minimum), and standard deviation (SD) of root mean square error (RMSE) values indicating performance of models in forecasting fatigue levels in low-back for 4 steps in the future.

Condition	Model Name	Average	Max	Min	SD
E	ARIMA	0.563	1.000	0.010	0.268
	ARIMA with External Features	0.862	3.338	0.219	1.003
	Prophet	0.615	1.079	0.337	0.241
	Prophet with External Features	1.537	3.538	0.564	0.905
	All Models	0.832	3.538	0.010	0.722
NE	ARIMA	1.036	1.936	0.250	0.452
	ARIMA with External Features	1.743	2.492	0.376	0.603
	Prophet	0.671	1.133	0.250	0.288
	Prophet with External Features	1.450	4.274	0.487	1.233
	All Models	1.103	4.274	0.250	0.782

**Table 4:** Performance of forecasting models expressed as root mean square error (RMSE) for forecasting fatigue levels in low-back up to 4 steps in the future for all study participants.

Participant Number	Model	Assistance	RMSE
1	ARIMA	E	0.511
		NE	1.936
	ARIMA with External Features	E	0.369
		NE	2.492
	Prophet	E	0.399
		NE	1.133
	Prophet with External Features	E	0.914
		NE	0.559
2	ARIMA	E	0.616
		NE	0.988
	ARIMA with External Features	E	0.451
		NE	0.376
	Prophet	E	0.626
		NE	0.523
	Prophet with External Features	E	0.564
		NE	4.274
3	ARIMA	E	0.471
		NE	1.000
	ARIMA with External Features	E	0.468
		NE	1.578
	Prophet	E	0.915
		NE	0.559
	Prophet with External Features	E	1.929
		NE	1.863
4	ARIMA	E	0.723
		NE	0.250
	ARIMA with External Features	E	1.519
		NE	1.450
	Prophet	E	0.337
		NE	0.250
	Prophet with External Features	E	1.181
		NE	2.136
5	ARIMA	E	1.000
		NE	1.224
	ARIMA with External Features	E	3.338
		NE	2.121
	Prophet	E	1.079
		NE	0.954
	Prophet with External Features	E	0.983
		NE	0.487
6	ARIMA	E	0.707
		NE	0.866
	ARIMA with External Features	E	0.306
		NE	1.965
	Prophet	E	0.446
		NE	0.817
	Prophet with External Features	E	3.538
		NE	0.585
7	ARIMA	E	0.538
		NE	1.323
	ARIMA with External Features	E	0.577
		NE	1.650
	Prophet	E	0.560
		NE	0.330
	Prophet with External Features	E	2.176
		NE	0.912
8	ARIMA	E	0.010
		NE	0.866
	ARIMA with External Features	E	0.219
		NE	2.088
	Prophet	E	0.568
		NE	0.656
	Prophet with External Features	E	1.081
		NE	1.645
9	ARIMA	E	0.500
		NE	0.866
	ARIMA with External Features	E	0.512
		NE	1.965
	Prophet	E	0.607
		NE	0.817
	Prophet with External Features	E	1.466
		NE	0.585

**Table 5:** Benefits of using exoskeletons depicted in terms of percent difference between with and without assistance conditions across forecasted fatigue levels at 20 trials.

Participant	ARIMA with External Features (% Benefits)	Prophet (% Benefits)
1	54.082	39.482
2	66.667	66.667
3	17.172	17.528
6	23.979	17.528
7	55.259	61.987
8	60.575	58.174
10	29.821	15.912
11	141.091	140.131
13	48.683	43.145
Mean	55.259	51.172
SD	34.394	36.557
Median	54.082	43.145

## Data Availability

The raw data supporting the conclusions of this article will be made available by the corresponding authors on reasonable request.
